# Supplementary material for: A live tumor fragment platform to assess immunotherapy response in core needle biopsies while addressing challenges of tumor heterogeneity
Source: J Transl Med. 2026 Jan 3;24:18. doi: 10.1186/s12967-025-07378-2 (PMC12763878; doi:10.1186/s12967-025-07378-2)
Supplement: Supplementary file 1 — Supplementary Material 1 [file 12967_2025_7378_MOESM1_ESM.docx]

# **SUPPLEMENTAL METHODS**

**Specimens**

*Mouse models:*

Experiments using murine models were performed in accordance with the Elephas Institutional Animal Care and Use Committee–approved protocol (see main Methods Female mice, approximately 6 weeks of age at arrival, were housed in a 12/12-hour light/dark cycle, provided ad libitum access to food and water, and supplied with cage-enrichment devices (eg, nesting tubes, exercise wheels). During tumor growth, mice were monitored for overall health through daily mass measurements and investigator observation. Tumors were measured twice weekly using calipers to determine length and width and to estimate volume (V = 0.5 L x W^2^).

*Syngeneic CT26:*

CT26 mouse colon carcinoma cells were cultured at 37 °C for ≤ 6 passages in RPMI-1640 medium (Gibco, 11975093) containing 10% fetal bovine serum (FBS) (Life Technologies, 10082147) and 1% penicillin-streptomycin (Gibco, 15140122).

*Humanized PDX:*

Engraftment was confirmed by flow cytometry analysis of human CD45^+^ cells (>10%) in the peripheral blood.

*Human tissue:*

Per treatment guidelines, PD-L1 expression was evaluated according to tumor type–specific thresholds. Tumors were classified as biomarker positive when PD-L1 tumor proportion score (TPS) was ≥50% for non-small cell lung cancer or when combined positive score (CPS) was ≥1 for head and neck squamous cell carcinoma. For MMR/MSI testing in colorectal and endometrial specimens, dMMR/MSI-High was classified as biomarker positive and pMMR/MSI-Low/MSS were classified as biomarker negative.

**Specimen preparation**

*Tumor resections:*

Resected tumors were attached to sample holders and embedded in a 3.8-4.0% w/v low-gelling temperature agarose (Sigma, A9045). The tumors were then carefully pushed through a cutting stage and cut into desired dimensions of resection LTFs (see main Methods). Resection LTFs were placed immediately into a 4 °C bath containing cutting media: RPMI-1640 medium (Gibco, 11875093) supplemented with either 10% heat-inactivated human serum (Sigma, H3667) or 10% heat-inactivated FBS (Life Technologies 10082147), 10 mM HEPES (Gibco, 1630080), 1X MEM (Sigma Aldrich, M7145), 1% sodium pyruvate (Gibco, 11360070), 1% Penicillin-Streptomycin (Gibco, 15140122), and 1% GlutaMAX (Gibco, 35050061). Resection LTFs were passed through a 420-µm filter and collected using a 200-µm filter before being resuspended in a 50-mL conical tube with cutting media. Resection LTFs were next counted by an automated digital camera employing proprietary software. Resuspended fragments were gently pipetted to mix before aliquoting into a 24-well plate based on the target LTF number per well. After encapsulation (see main Methods) the plate was imaged on the automated digital microscope to calculate the tissue volume present in each well. Resection LTFs were then maintained at 37 °C and 5% CO_2_ throughout experimentation.

*Core Needle Biopsies:*

CNBs of various gauges (see main Methods) were measured for length, embedded in agarose solution, and chilled on ice for 5 minutes to allow the agarose to solidify, before being cut into slices with a thickness of 300 µm using an automated, proprietary cutting device. CNB LTFs were counted manually, equally distributed into the wells, and 300 µL of a hydrogel was added to each well. Fragments embedded in Elephas’ proprietary hydrogel were exposed to a 395-nm UV light to polymerize and washed three times with Dulbecco’s phosphate buffered saline. After hydrogel polymerization, 500 µL of culture media containing a treatment was added to each well with tissue, and the plate was imaged on the automated digital camera to calculate the tissue volume present in each well. Specimens were maintained at 37 °C and 5% CO_2_ throughout experimentation.

**Treatments**

αCD3/αCD28 stimulation was performed with ImmunoCult^TM^ Human CD3/CD28 T Cell at a final concentration of 25 µL/mL, except in select experiments (see Figure 5), for which 100 µL/mL was used. This higher concentration was based on a pilot study, which found the effect of αCD3/αCD28 stimulation reaches a plateau at this level (data not shown). IgG and ICI antibodies were used at a concentration of 50 µg/mL, and a control well treated only with a human IgG antibody was used at the final concentration of 50 µg/mL immediately after plating. The control for αCD3/αCD28 and αPD-1 was a human IgG4 antibody (RecombiMAb human IgG4 [S288P, CP147], αPD-L1 was a human IgG1 antibody (RecombiMAb human IgG1 [N297A], CP171) and αPD-1 and αCTLA-4 combination treatment was a combination of the human IgG4 antibody and a human IgG1 antibody (RecombiMAb human IgG1 isotype control, CP174). Exceptions to IgG matching include Colon 4834, Esophageal 4798, and Kidney 5023 where control was IgG4, and Kidney 5327 where control was IgG4+IgG1.

**Assays**

*Viability and cytotoxicity assays*:

The CCK8 reagent, WST-8 (water-soluble tetrazolium salt) was diluted with culture medium and added to the culture well to obtain a final concentration of 0.1X at the start of the assay. At a specified time point, 60 µL of conditioned media containing the reagent was collected from a culture well into a clear, flat-bottom 96-well plate, and absorbance was measured at 450 nm using a plate reader (BioLegend, 423555). Media containing CCK8 were used as a blank for the assay, and relative viability was reported as the change in absorbance per hour.

LDH was measured using the LDH-Glo^TM^ Cytotoxicity Assay kit. Conditioned media from a culture well were diluted 1:20 in the LDH storage buffer (200 mM Tris-HCl [Thermo Fisher Scientific, J67501-AK], 10% Glycerol [Acros Organics, 327255000], and 1% BSA [Sigma Aldrich, A2058]) at the time of collection and frozen at -80 ^o^C for a minimum of 1 day before processing. Samples were thawed on ice and centrifuged before use. LDH Standard was diluted to the concentration of 32 mU/mL, and serial dilutions were prepared with LDH storage buffer. Samples were added to a 96-well assay plate and mixed with LDH Detection Enzyme Mix and Reductase Substrate. The plate was incubated at room temperature for 30-60 minutes, protected from light, before being read for luminescence on a plate reader (PerkinElmer, EnSpire Alpha).

*Optical Coherence Microscopy imaging:*

A custom-designed optical coherence microscopy (OCM) system and accompanying software system (Elephas) was used to acquire time-resolved, volumetric dynamic OCM (dOCM) data for viability assessment. The time series of each cross section was first registered to the center frame. For each pixel in the time series of volumetric OCM intensity I(x, y, z, t), fast Fourier transform was performed to obtain the power spectrum P(x, y, z, f). Intensity images were also acquired on the same system for structural analyses. Images were acquired every 24 hours for the same tumor specimen over 3 days of culture.

**Cytokine profiling:**

Using proprietary imaging software, the total fragment volume in each well was calculated to permit normalization of cytokine concentrations. Conditioned media collected from individual culture wells at defined time points were assessed. The cytokine concentrations from samples were interpolated from a standard curve generated for each analyte (see main Methods). Replicate measurements were averaged unless otherwise noted. A cumulation of analyte concentrations was calculated to account for the portion of the analyte removed from the well at earlier time points whenever the earlier concentrations were assayed. For example, the analyte concentration at 4 hours was multiplied by the fractional supernatant draw relative to the total supernatant volume in the well and added to the concentration at 24 hours (the next time point) as shown in the equation below:

$$Cumulative {Conc}_{analyte}=Conc_{analyte}^{24hr}+Conc_{analyte}^{4hr}*\frac{100 \mu L}{500 \mu L}$$

Samples with multiple replicates in either the αPD-1 or IgG-treated group in the cross-well comparisons had their cumulative concentrations for each analyte averaged across all replicates. The sequential treatment configuration compared the cumulative concentrations from each replicate well separately.

**Histology:**

Fixed LTFs (see main Methods) were placed in a custom-designed 3D printed mold. This procedure was designed to optimize the spatial arrangement of tumor fragments to produce a representative sampling of the tumor histology. Sections were mounted to slides using a tissue flotation water bath. Slides were then stained with hematoxylin and eosin (H&E) or processed for immunohistochemistry (IHC) and immunofluorescence (IF) (see main Methods). Individual channels of mIF images were adjusted using Adobe Photoshop to reduce the effects of exposure-related intensity non-uniformities introduced during image acquisition, and a global curve adjustment layer was applied to enhance overall image contrast. These adjustments were made solely to improve visual presentation and mitigate stitching-induced non-uniformities and were not used for any quantitative analyses.

**Statistical analyses:**

For cytokine analysis, conditioned media were collected at the ~4-, ~20- and ~48-hour time points. The rate of change (slope) of cumulative cytokine concentration (CC) was calculated for ICI and IgG treatment phases, as shown below.

$$\mathrm{Slope}_{IgG}=\frac{CC_{20 hour}-CC_{4 hour}}{\Delta_{time}}$$

$$\mathrm{Slope}_{ICI}=\frac{CC_{48 hour}-CC_{20 hour}}{\Delta_{time}}$$

The fold change of slope (Slope_FC_) was then calculated, as shown below. If multiple replicate wells were tested for a given specimen, the maximum fold change of slope was reported.

$$\mathrm{Slope}_{FC}=\frac{{Slope}_{ICI}}{{Slope}_{IgG}}$$

For resections, ICI-induced change in cytokine concentrations was determined by the difference in cumulative concentrations between ICI- and IgG-treated wells (Delta), as shown below:

$$\Delta_{analyte}=CC_{analyte}^{\alpha PD1}-CC_{analyte}^{IgG}$$

To normalize the varying scale of Delta values across analytes and identify a potential responder to treatment, a trimmed-sample approach was applied. Calculations of the trimmed- sample statistics occurred in two stages and leveraged the median (eg, 50th percentile, E[X]) and median absolute difference (MAD) to describe the central tendency and spread of the population, respectively. The equation for MAD is shown below, where E denotes a median operator:

$$MAD=E[\left| X-E\left[ X \right] \right|]$$

In the first stage, the median and MAD values were calculated using the full complement of samples. These initial values were used to identify inliers, with the definition of an inlier as any sample that falls within two MAD values of the median (ie, median - 2*MAD $\leq$ inlier $\leq$ median + 2*MAD). Using only the inliers, the median and MAD values were then recomputed to generate the trimmed statistics. The trimmed data were then transformed to modified Z-scores (Mod. Z, 25) by the equation below:

$$Mod. Z= \frac{X-E[X]}{MAD}*0.6745$$

This method allows for easier identification of upregulated cytokines through outliers relative to cytokines that exhibit no change or a decrease with αPD-1 treatment. The Mod. Z transformation differs from a traditional Z-score transformation in that the central tendency and spread terms are derived from the median and MAD values, rather than the mean and standard deviation. This approach is known to be more robust to extreme outliers due to the reliance on the median and MAD and is better optimized for outlier detection than a traditional Z-score method.

Agglomerative hierarchical clustering was performed on the data, clustering both the individual samples (columns) and the analytes (rows) by Ward’s method using TIBCO Spotfire™ software. The heatmap for changes in cytokine levels was generated using the Delta values for each analyte after statistical trimming and Mod. Z transformation of the cytokine dataset. Mod. Z-scores of -10 and 10 were selected as the lower and upper saturation points, respectively, as any value beyond this range already indicates meaningful analyte regulation (see main Methods).
