# Supplementary material for: A live tumor fragment platform to assess immunotherapy response in core needle biopsies while addressing challenges of tumor heterogeneity
Source: J Transl Med. 2026 Jan 3;24:18. doi: 10.1186/s12967-025-07378-2 (PMC12763878; doi:10.1186/s12967-025-07378-2)
Supplement: Supplementary file 2 — Supplementary Material 2 [file 12967_2025_7378_MOESM2_ESM.docx]

#

# **SUPPLEMENTAL FIGURES**

# **
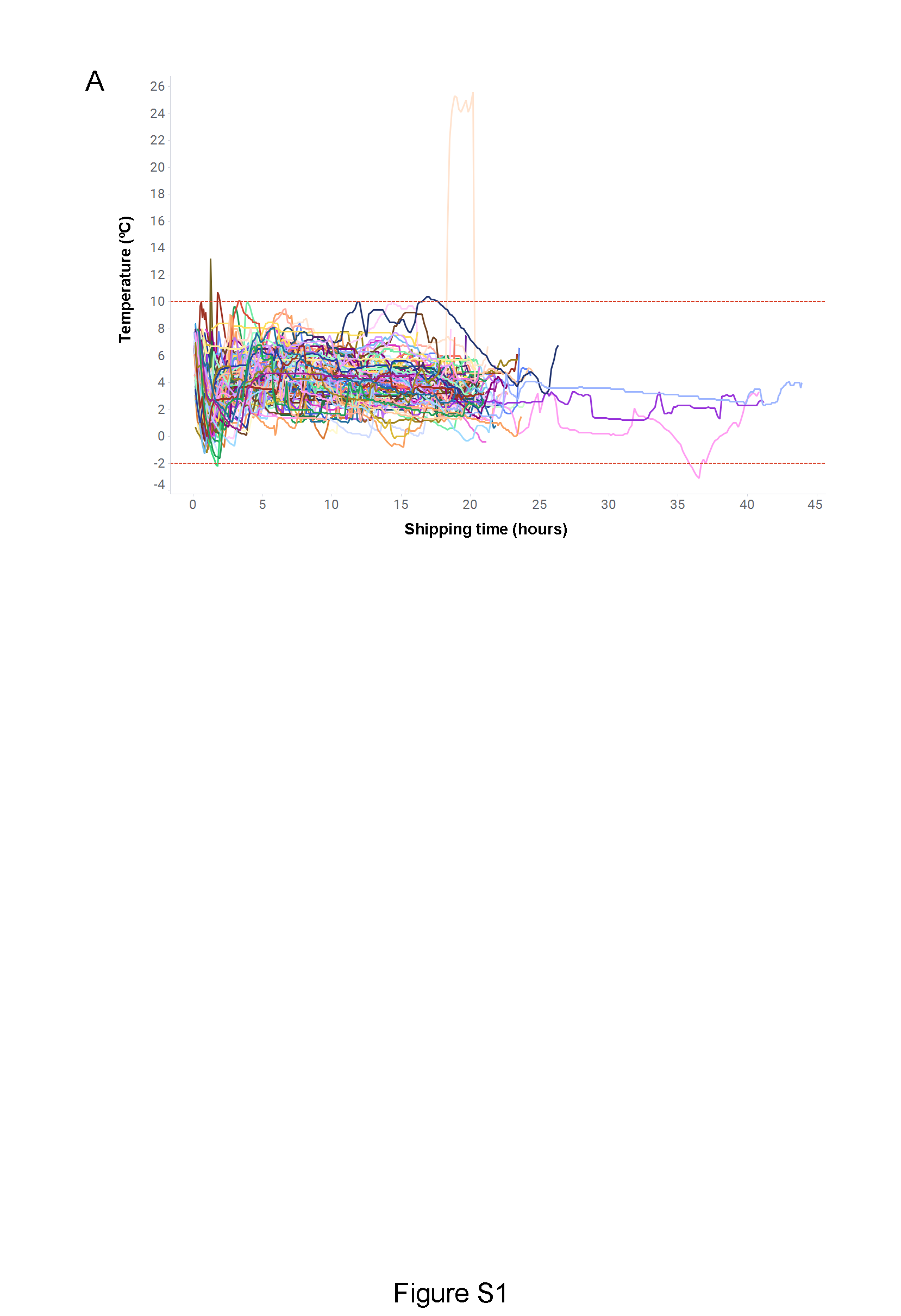
Fig S1.** Internal temperature of NanoCool shipping system vs. shipping time for 118 tumor specimens collected consecutively shows tight temperature regulation during overnight shipping of CNBs from diverse clinical sites.

**
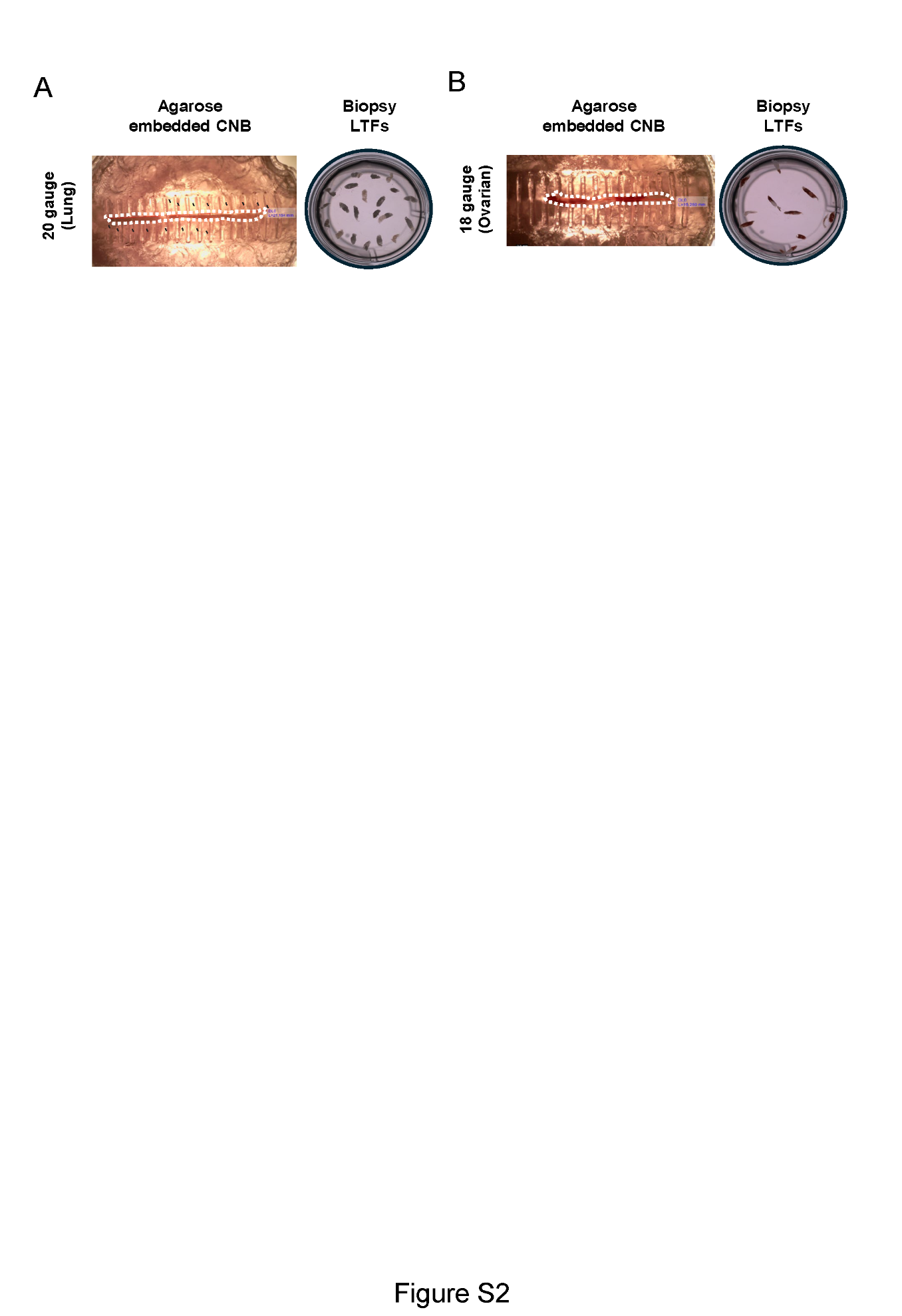
**

**Fig S2.** Representative images showing a lung tumor CNB specimen from a 20-gauge biopsy needle (**A**) and an ovarian tumor CNB specimen from an 18-gauge biopsy needle (**B**) embedded in agarose in preparation for cutting (left) and resulting CNB LTFs after cutting (right). Note that vertical marks in the agarose gel are an artifact of the agarose mold and not the result of cuts made through the CNB.


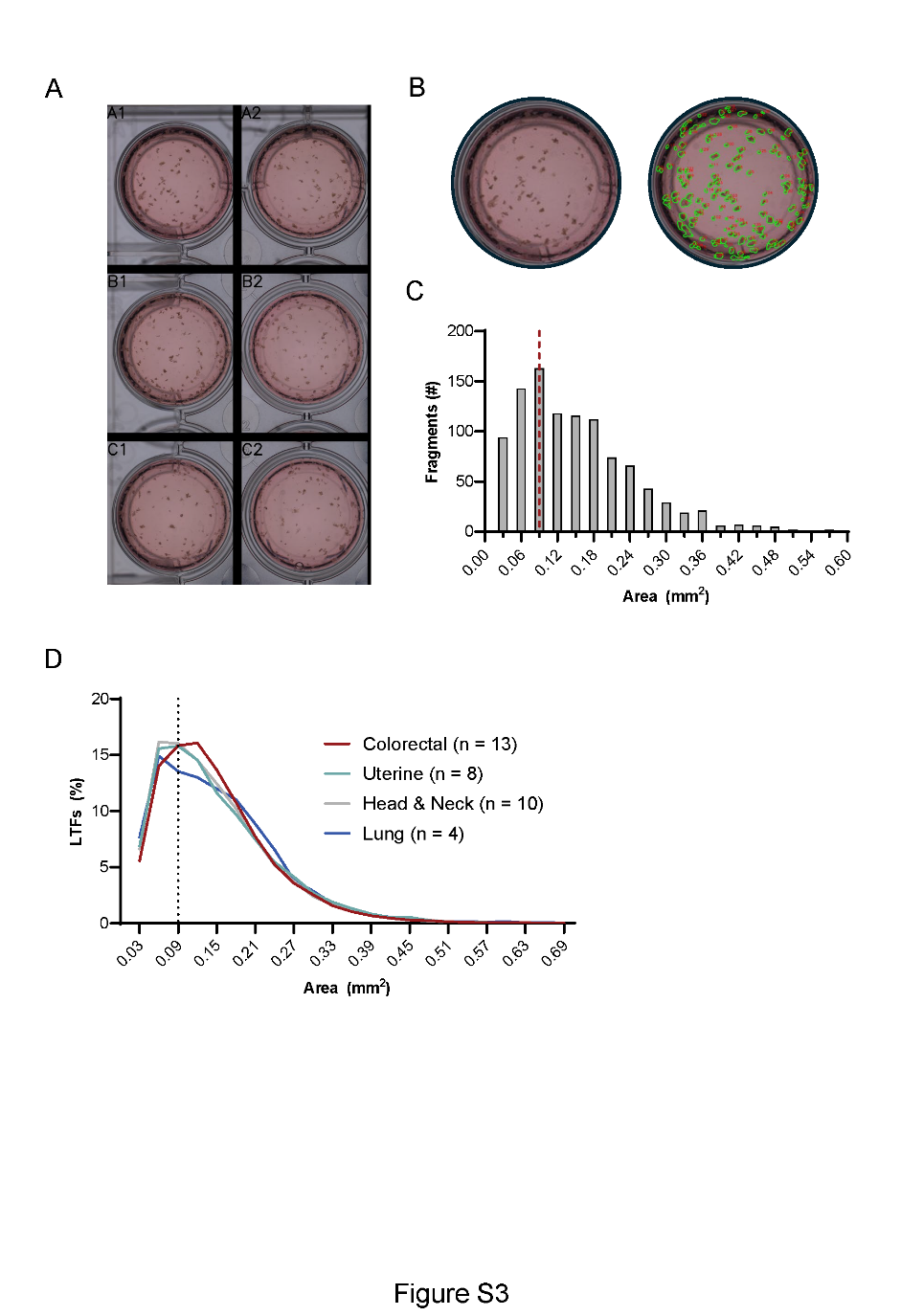


**Fig S3.** Resected human tumors were cut using a proprietary cutting instrument that can cut tumor tissue automatically at user-specified length, width, and depth dimensions. Cutting tumors to 300 µm (length) x 300 µm (width) x 300 µm (depth) in size generates defined tissue areas across fragments that can then be randomly allocated to sample wells for treatment comparisons. **(A)** Resection LTFs were manually distributed amongst 6 wells of a culture plate after cutting. An enlarged view of one of the wells (A1, left) and outlines of fragment area used for area calculations (right) are shown. **(C)** The number of fragments binned to a discrete range of areas are reported for the entire resected tumor. The dotted line represents the expected surface area for one face of a 300 µm x 300 µm x 300 µm cut fragment. **(D)** Histogram presenting the binned areas for fragments of resected human tumors cut to 300 µm × 300 µm × 300 µm (colorectal, n=13; endometrial, n=8; head and neck, n=10; and lung, n=4).

**
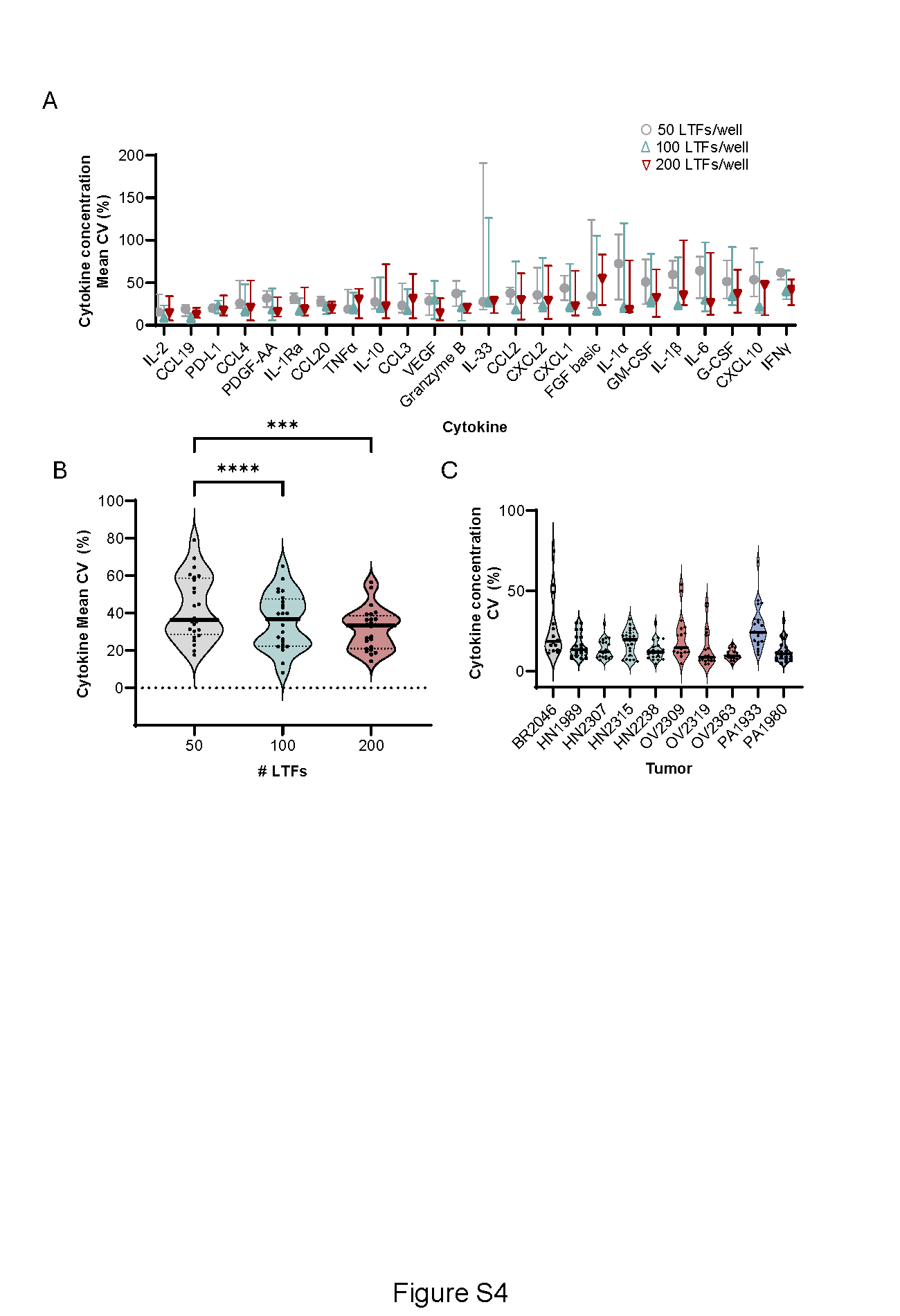
**

**Fig S4. (A)** Effect of LTF number (~50, ~100, or ~200 LTFs per well) on variability [mean CV (%]) of cytokine induction (n=24 cytokines) following 24 hours of αCD3/αCD28 stimulation in 5 human tumor resections (lung, n=1; ovarian, n=1; colorectal, n=1; head and neck, n=2), each including 3 replicates reported per cytokine (3 replicates per tumor specimen). (**B)** Violin plot of mean CV (%) across all cytokines for 50, 100, and 200 fragments is shown with an observed decrease in the spread of CV with increase in number of fragments. **(C)** Variability (CV [%]) in cytokine concentrations across 5 replicate wells, each containing ~200 resection LTFs cut from 10 human tumors (pancreatic, n=2; head and neck, n=4; ovarian, n=3; triple-negative breast, n=1) stimulated with αCD3/αCD28 for 48 hours. *** *P* < 0.001 **** *P* < 0.0001.


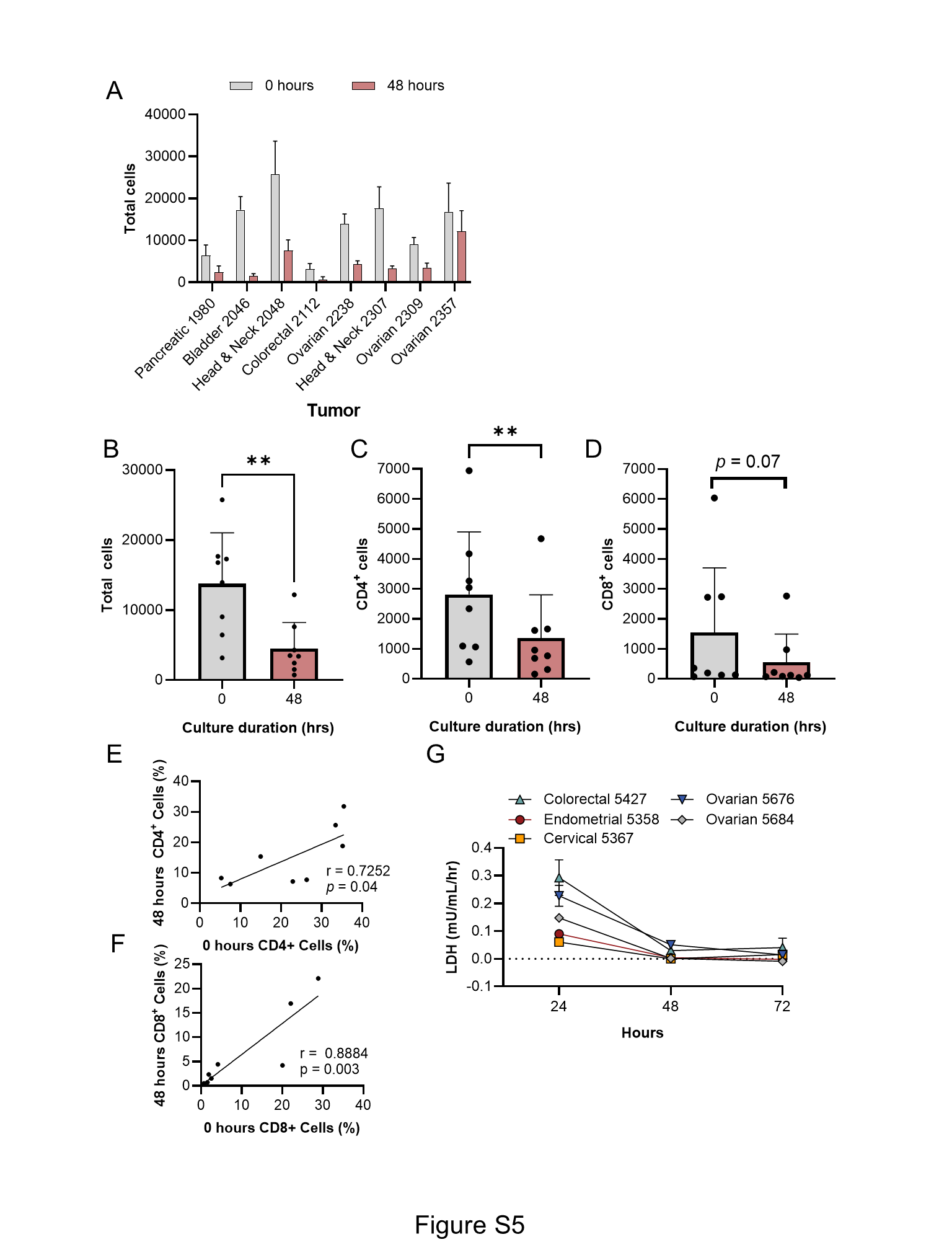


**Fig S5**. **(A)** Comparison of the total cells (as assessed by nuclear count) between 0 and 48 hours of culture. Summarized data for all specimens across culture duration for total cells **(B)**, CD4^+^ cells **(C)**, and CD8^+^ cells **(D)**. The percentage of CD4^+^ cells **(E)** and CD8^+^ cells **(F)** show correlation between hours 0 and 48. **(G)** Changes in cytotoxicity (LDH assay) over 72 hours of ex vivo culture in LTFs derived from 5 human tumor resections shows a decrease in cytotoxicity between 24 and 48 hours, followed by stabilization between 48 and 72 hours of culture.

*
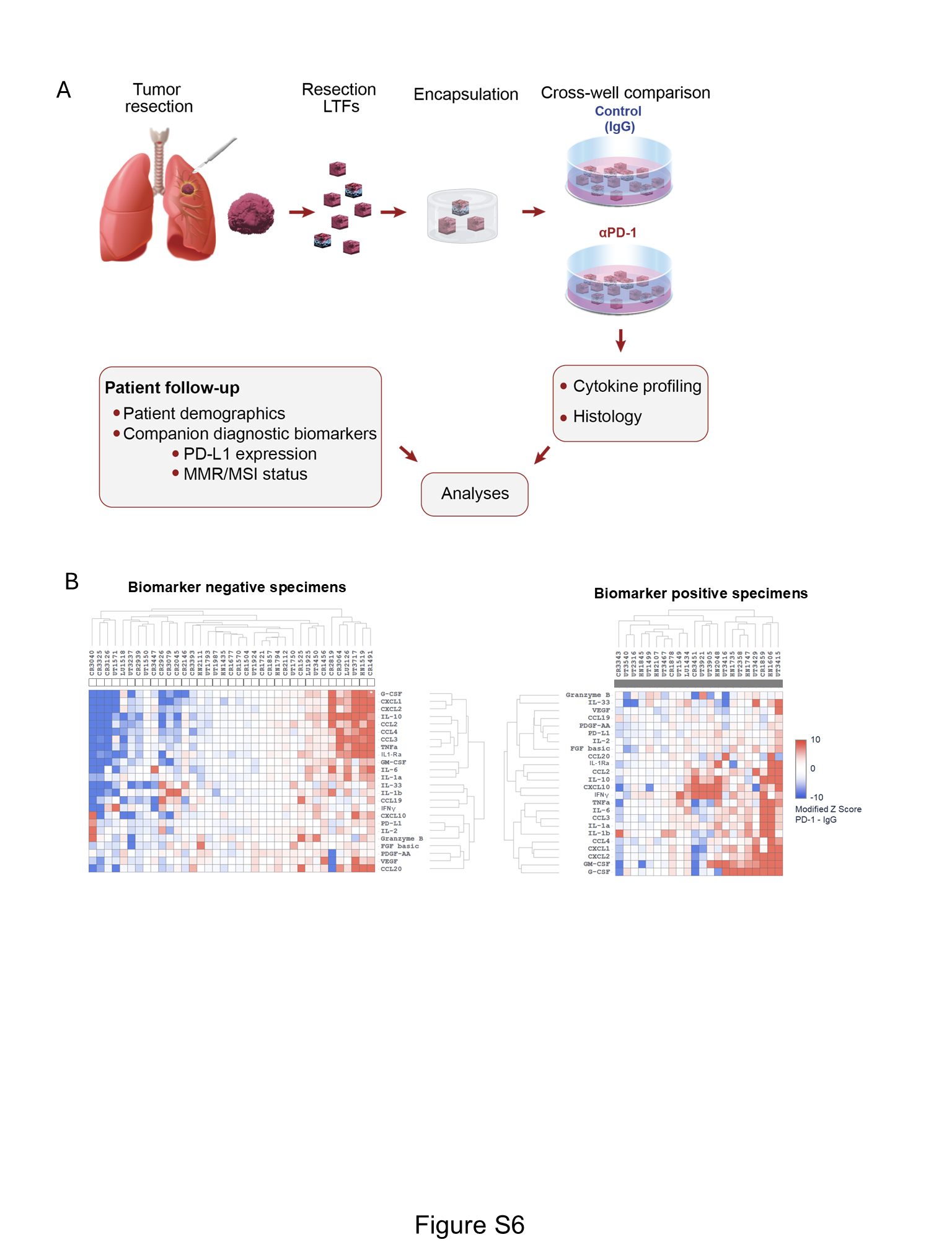
***Fig S6. (A)** Schematic showing the methods used for creating resection LTFs to assess cytokine response to anti–PD-1 treatment in PD-L1/MMR/MSI-positive (PD-L1^+^/dMMR/MSI-high) and PD-L1/MMR/MSI-negative (PD-L1^-^/pMMR/MSS) specimens. **(B)** Unsupervised hierarchical clustering of cytokine profiles from patient tumor resections using modified Z-scores of the difference in cytokine concentrations between the ICI- and IgG-treated groups; PD-L1/MMR/MSI-positive and PD-L1/MMR/MSI-negative specimens are presented separately. *p<0.05; ** p < 0.01.


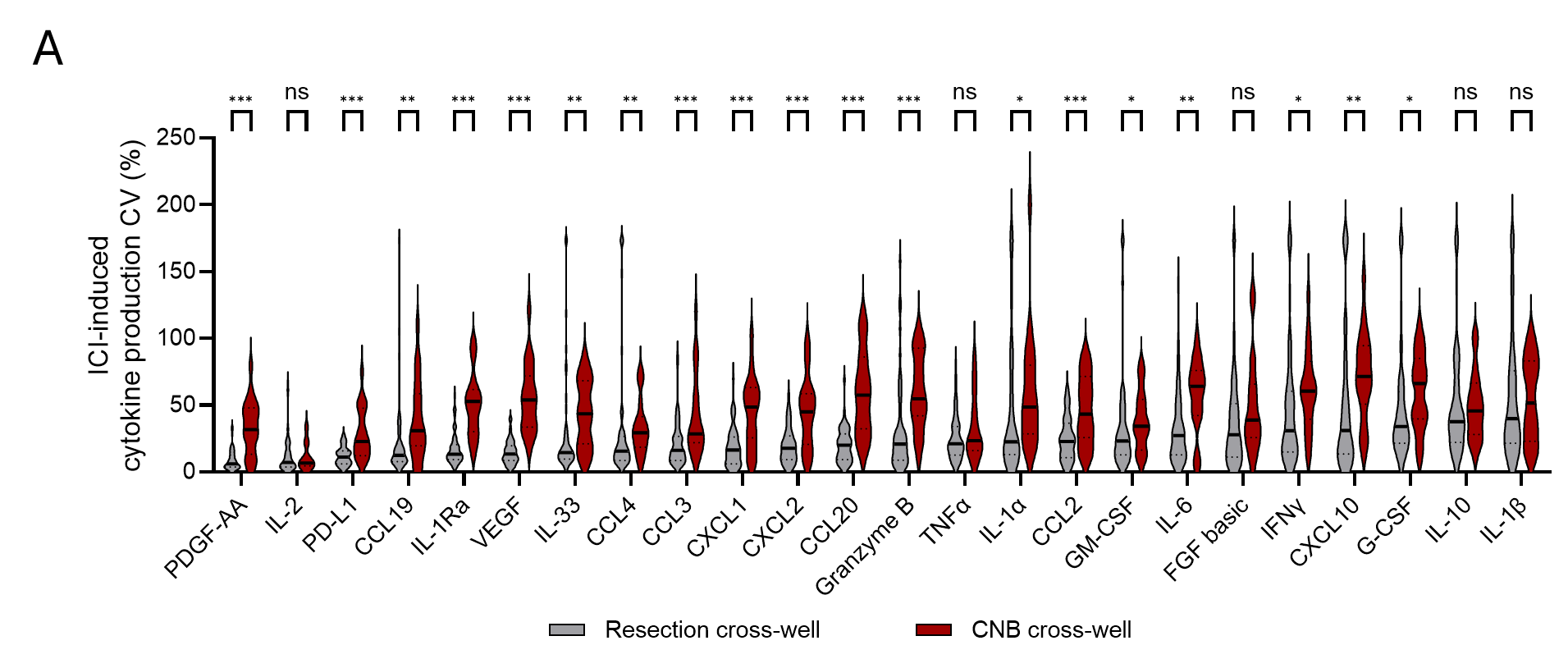


**Fig S7.** Variance shown as CV (%) of ICI-induced cytokine production in resection and CNB cross-well experiments represented for each cytokine measured. * *p* < 0.05, ** *p* < 0.005, *** *p* < 0.001.


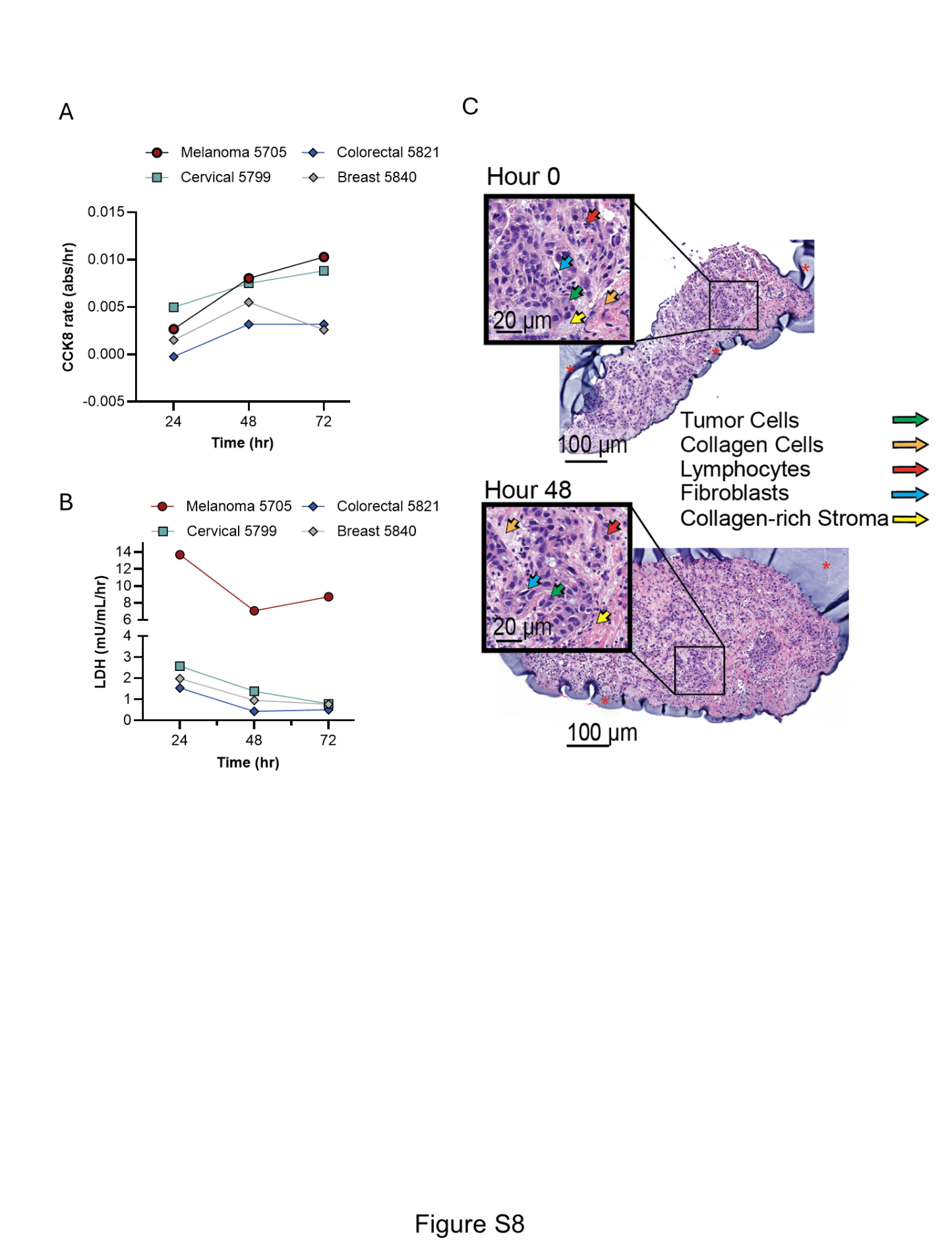


**Fig S8. (A)** CCK8 analysis of CNB LTFs derived from 4 human tumor resections demonstrates maintenance of viability over 72 hours of culture. **(B)** Cytotoxicity evaluation (LDH assay) over 72 hours of ex vivo culture in LTFs derived from 4 human tumor CNBs shows a decrease in cytotoxicity between 24 and 48 hours, followed by stabilization between 48 and 72 hours of culture, similar to what was shown in resection LTFs. **(C)** H&E-stained sections of two independent LTFs from a human liver cancer (Liver 5605) biopsy LTF encapsulated in Elephas hydrogel and cultured for 0 (upper) or 48 (lower) hours. Both 0-hour and 48-hour CNB LTFs show islands of carcinoma cells (green arrow) within sparse stroma (matrix-enriched stroma, yellow arrow; collagen-rich stroma, orange arrow), lymphocytes (red arrow) and fibroblasts (blue arrow).


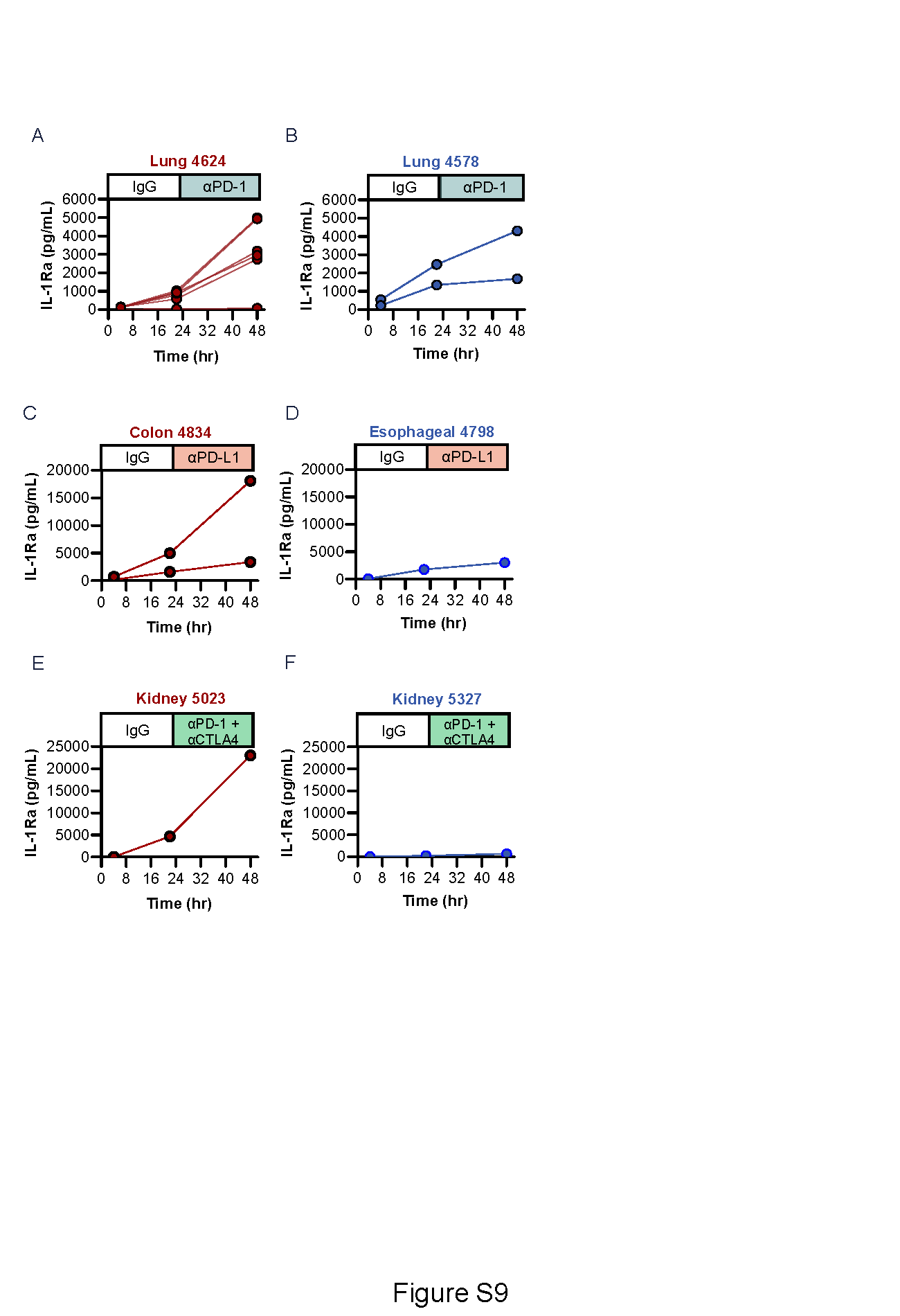


**Fig S9.** IL-1Ra represents a cytokine for which little to no change in cytokine production rate was observed between the IgG and ICI treatment phases for all specimens profiled. IL-1Ra measured at 4, 24 and 48 hours for specimens which showed an increase in IFNγ and CXCL10 induction following ICI treatment (αPD-1, **[A]**; αPD-L1,**[C];** αPD-1 + αCTLA-4, **[E]**) and specimens that showed no change in IFNγ and CXCL10 induction following ICI treatment (αPD-1, **[B]**; αPD-L1, **[D];** αPD-1 + αCTLA-4, **[F]**). IL-1Ra shows robust concentrations with a smaller induction rate during the IgG and ICI treatment phases.

# **SUPPLEMENTAL TABLES**

**Table S1.** List of cytokines assayed. Cytokines denoted with an asterisk (*) were often not detected and were therefore excluded from analysis.

| **Analytes in the 30-plex Luminex assay** | |
| --- | --- |
| **CCL19/MIP-3β** | **Lymphotoxin-α/TNF-β*** |
| **CCL2/JE/MCP-1** | **IL-10** |
| **CCL20/MIP-3α** | **IL-12 p70*** |
| **PD-L1/B7-H1** | **IL-15*** |
| **CCL3/MIP-1α** | **IL-1β/IL-1F2** |
| **CCL4/MIP-1β** | **IL-1Ra/IL-1F3** |
| **CD40 ligand/TNFSF5*** | **IL-2** |
| **CXCL1/GROα/KC/CINC-1** | **IL-33** |
| **CXCL10/IP-10/CRG-2** | **IL-6** |
| **CXCL2/GROβ/MIP-2/CINC-3** | **PDGF-AA** |
| **EGF*** | **TNF-α** |
| **IFNγ** | **VEGF** |
| **Flt-3 Ligand/FLT3L*** | **FGF basic/FGF2/bFGF** |
| **G-CSF** | **IL-1α/IL-1F1** |
| **GM-CSF** | **Granzyme B** |

**Table S2.** List of antibodies used for IHC and immunofluorescence experiments.

| **Antibody** | **Clone** | **Manufacturer and catalog number** |
| --- | --- | --- |
| Actin, smooth muscle (SMA) | 1A4 | Cell Marque #202M |
| Anti-CD3 | 2GV6 | Ventana Medical Systems #790-4341 |
| Anti-CD4 | SP35 | Ventana Medical Systems #790-4423 |
| Anti-CD8 | SP57 | Ventana Medical Systems #790-4460 |
| Anti-CD20 | L26 | Ventana Medical Systems #760-2531 |
| Anti-CD68 | KP-1 | Ventana Medical Systems #790-2931 |
| Anti-pan keratin | AE1/AE3 & PCK26 | Ventana Medical Systems #760-2595 |

| **Patient Characteristics** | | | |
| --- | --- | --- | --- |
|  |  | **N** | **%** |
| Sex | M | 21 | 35.6% |
|  | F | 30 | 50.8% |
|  | Unknown | 8 | 13.6% |
| Age | Range (years) | 37-88 | NA |
|  | 30-40 | 2 | 3.4% |
|  | 41-50 | 9 | 15.3% |
|  | 51-60 | 8 | 13.6% |
|  | 61-70 | 15 | 25.4% |
|  | 71-80 | 9 | 15.3% |
|  | >80 | 7 | 11.9% |
|  | Unknown | 9 | 15.3% |
| Race | White | 41 | 68.3% |
|  | Black | 6 | 10.2% |
|  | American Indian or Alaska Native | 1 | 1.7% |
|  | Unknown | 11 | 18.6% |
| **Tumor type** | **Lung** | 10 | 16.9% |
|  | **Head & Neck** | 4 | 6.8% |
|  | **Colorectal** | 25 | 42.4% |
|  | **Uterine** | 20 | 33.9% |
|  | **Primary** | 42 | 71.2% |
|  | **Secondary** | 17 | 28.8% |
| **Companion diagnostic biomarker status** | **Positive** | 22 | 37.3% |
|  | PD-L1 | 7 | 31.8% |
|  | MSI | 15 | 68.2% |
|  | **Negative** | 37 | 62.7% |
|  | PD-L1 | 7 | 18.9% |
|  | MSI | 30 | 81.1 |
| Clinical stage | 1 | 5 | 8.5% |
|  | 2 | 6 | 10.2% |
|  | 3 | 9 | 15.3% |
|  | 4 | 6 | 10.2% |
|  | Unknown | 33 | 55.9% |

**Table S3.** Selected patient characteristics, including demographics, diagnoses, companion diagnostic biomarker status, clinical stage, and treatments for the biomarker resection LTF study.

| **IO Treatment** | | | |
| --- | --- | --- | --- |
|  |  | **N** | **%** |
| Neoadjuvant IO | Yes | 2 | 3.4% |
|  | No | 20 | 33.9% |
|  | Unknown | 37 | 62.7% |
| Adjuvant IO | Yes | 4 | 6.8% |
|  | No | 35 | 59.3% |
|  | Unknown | 20 | 33.9% |
| Chemotherapy | Yes | 7 | 11.9% |
|  | No | 21 | 35.6% |
|  | Unknown | 31 | 52.5% |

**Table S4.** Cytokines showing increased rate of production in ICI-treated compared with IgG-treated LTFs from PD-L1/MMR/MSI-positive and PD-L1/MMR/MSI-negative specimens with statistical comparisons. GM-CSF, IFNγ, and CXCL10 all show significantly increased rates of cytokine production in the PD-L1/MMR/MSI-positive compared with PD-L1/MMR/MSI negative specimens.

| **Cytokines** | **PD-L1/MMR/MSI- positive rate** | **PD-L1/MMR/MSI- negative rate** | ***p*-value** |
| --- | --- | --- | --- |
| GM-CSF | 0.41 | 0.03 | 0.0003 |
| IFNγ | 0.36 | 0.03 | 0.001 |
| CXCL10 | 0.32 | 0.08 | 0.03 |
| G-CSF | 0.36 | 0.14 | 0.055 |
| IL-1β | 0.32 | 0.11 | 0.081 |
| CCL2 | 0.18 | 0.08 | 0.407 |
| CXCL2 | 0.18 | 0.11 | 0.455 |
| IL-1α | 0.18 | 0.11 | 0.455 |
| TNFα | 0.23 | 0.14 | 0.477 |
| CCL19 | 0.00 | 0.05 | 0.524 |
| IL-2 | 0.00 | 0.05 | 0.524 |
| IL-33 | 0.09 | 0.05 | 0.624 |
| CCL20 | 0.09 | 0.16 | 0.697 |
| CCL3 | 0.09 | 0.14 | 0.702 |
| CXCL1 | 0.18 | 0.14 | 0.715 |
| IL-10 | 0.23 | 0.16 | 0.731 |
| CCL4 | 0.14 | 0.11 | 1.0 |
| FGF basic | 0.05 | 0.05 | 1.0 |
| Granzyme B | 0.05 | 0.08 | 1.0 |
| IL-1Ra | 0.14 | 0.14 | 1.0 |
| IL-6 | 0.18 | 0.16 | 1.0 |
| PD-L1 | 0.00 | 0.03 | 1.0 |
| PDGF-AA | 0.00 | 0.00 | 1.0 |
| VEGF | 0.05 | 0.05 | 1.0 |

**Table S5.** Estimates for the target total tissue per well for resection LTFs cut to 300-µm cubes and CNB LTFs cut at a 90º or 20º angle from samples collected by various needle gauges.

| **LTF characteristics** | | | | | | | | | | | |
| --- | --- | --- | --- | --- | --- | --- | --- | --- | --- | --- | --- |
|  | | **Shape** | **L**  **(mm)** | **W**  **(mm)** | **D**  **(mm)** | **Diameter**  **(mm)** | **Long axis**  **(mm)** | **Short axis**  **(mm)** | **Volume per fragment**  **(mm^3^)** | **# frags/well** | **Total tissue volume (mm^3^)** |
| **Resections** | | | | | | | | | | | |
| **Scored and cut** | | **Cuboid** | **0.3** | **0.3** | **0.3** |  |  |  | **0.027** | **200** | **5.40** |
| **Biopsies** | | | | | | | | | | | |
| **90° cut** | **12 g** | **Circular** |  |  | **0.3** | **2.2** |  |  | **1.140** | **5** | **5.70** |
|  | **14 g** | **Circular** |  |  | **0.3** | **1.6** |  |  | **0.603** | **9** | **5.43** |
|  | **16 g** | **Circular** |  |  | **0.3** | **1.2** |  |  | **0.339** | **16** | **5.43** |
|  | **18 g** | **Circular** |  |  | **0.3** | **0.8** |  |  | **0.166** | **33** | **5.49** |
|  | **20 g** | **Circular** |  |  | **0.3** | **0.6** |  |  | **0.085** | **64** | **5.43** |
| **20° cut** | **12 g** | **Oval** |  |  | **0.3** |  | **6.11** | **2.07** | **2.980** | **2** | **5.96** |
|  | **14 g** | **Oval** |  |  | **0.3** |  | **4.44** | **1.51** | **1.580** | **3** | **4.74** |
|  | **16 g** | **Oval** |  |  | **0.3** |  | **3.33** | **1.13** | **0.887** | **6** | **5.32** |
|  | **18 g** | **Oval** |  |  | **0.3** |  | **2.33** | **0.80** | **0.439** | **12** | **5.27** |
|  | **20 g** | **Oval** |  |  | **0.3** |  | **1.66** | **0.56** | **0.219** | **25** | **5.47** |
